# State licenses for medical marijuana dispensaries: neighborhood-level determinants of applicant quality in Missouri

**DOI:** 10.1186/s42238-024-00223-1

**Published:** 2024-03-26

**Authors:** David M. Yaskewich

**Affiliations:** Southeast Missouri State University, One University Plaza; Mailstop #5845, Cape Girardeau, MO 63701 USA

**Keywords:** Cannabis, Dispensaries, Licensing, Marijuana, Missouri, State government

## Abstract

**Background:**

When state governments impose quotas on commercial marijuana licenses, regulatory commissions use an application process to assess the feasibility of prospective businesses. Decisions on license applications are often met with formal appeals and legal challenges from rejected applicants. Although prior research has examined substate disparities in the availability of marijuana dispensaries, less attention has been given to the quality of license applications. The present study analyzed the relationship between neighborhood-level characteristics and the quality of prospective dispensary businesses.

**Methods:**

During Missouri’s first applicant pool for medical marijuana dispensaries in 2019, a total of 606 census tracts contained the location site of at least one dispensary applicant. Using data from the Missouri Department of Health and Senior Services and the American Community Survey, fractional and binary logistic regression models were used to estimate the relationship between census-tract characteristics and application outcomes.

**Results:**

License applications received higher evaluation scores when proposed dispensary sites were in census tracts with greater population densities and no majority in racial/ethnic composition. Census tracts with poorer socioeconomic conditions attracted a disproportionate share of low-scoring applicants from the bottom quartile of scores. These effects were stronger for certain application subsections, particularly those assessing the quality of an applicant’s business plan and on-site security.

**Conclusions:**

Some communities tend to attract prospective license holders who possess better quality resources, business practices, and industry experience. State disparities in commercial licensing requirements and application processes may lead to the inequities in legal product access found in some prior studies.

Within US states where marijuana is legal, multiple barriers may prevent businesses from entering regulated markets. Due to ongoing prohibition by the federal government, which has classified marijuana as a Schedule I controlled substance (US Drug Enforcement Agency 2023), many banks have not provided traditional services to marijuana businesses, such as commercial loans, business accounts, or electronic fund transfers (Owens-Ott 2020; Taylor et al. 2016). Another entry barrier can include the complexity of regulations on licensed businesses, which may require a team of professionals with expertise in cannabis, law, and accounting. Many states impose regulations related to product safety, packaging and labeling, on-site security, inventory management, and financial accounting and reporting. State governments also collect taxes from marijuana sales and can impose fines on establishments that violate laws and regulations. The regulatory burden on licensed businesses has been reflected by the prevalence of unlicensed sellers, who may not comply with the same rules (Nicholas et al. 2021), in some cities and states. In particular, past studies have observed a strong presence of unlicensed sellers in California (Firth et al. 2022; Goldstein and Sumner 2022; Unger et al., 2020).^1^

Efforts to develop regulated markets also have encountered disparities in legal market participation. Site locations for marijuana dispensaries have been found to follow an uneven geographic distribution and concentrate in communities with more commercial retail activity (Boggess et al. 2014; Novak et al. 2021; Thomas and Freisthler 2016), socioeconomic deprivation (Amiri et al. 2019; Firth et al. 2020; Matthay et al., 2022b; Morrison et al. 2014; Tabb et al. 2018), and residents belonging to racial or ethnic minorities (Shi et al. 2016). Some reports also have expressed caution about the exclusion of certain segments of the population, particularly those who were adversely impacted by prior prohibition, from experiencing the economic benefits generated from legal sales (Bailey 2021; Marijuana Business Daily 2022). Two points of interest have included the use of marijuana-related tax revenue and underrepresentation of specific groups in the ownership of licensed marijuana businesses. Concerns about these inequities have influenced policies directed at the supply-side of the market. Several states have created social equity programs that provide grants and technical assistance to business applicants from targeted groups, such as racial and ethnic minorities, residents of low-income communities, or individuals with a prior marijuana-related conviction (Silverman et al. 2023).

Legislative bodies and regulatory agencies also can influence the retail landscape via commercial licensing policies. The strictness of regulations on business licensing can vary significantly across states. For example, among states that have legalized medical marijuana, Wang and Wilson (2022) observed that early-adopting states imposed relatively lenient regulations by distributing a higher volume of licenses and permitting a wider range of license categories beyond cultivation and retail activities.^2^ Some states have used formal scoring systems for license applications, which enable regulatory bodies to prioritize applicants by placing greater weight on certain attributes (Hannah et al. 2023). For example, scoring systems can vary according to the relative importance given to an applicant’s business plan, on-site security, or relevant experience.

While states have attempted to achieve equity in the allocation of commercial licenses, prior studies have not examined whether applicant quality differs across communities. The absence of an observable and direct measurement of applicant quality, which is a latent variable, poses a challenge for empirical analyses. If applicant quality is unevenly distributed, legal markets may continue to under- or overserve some regions due to local deficiencies in the financial capital, experience, or entrepreneurial skills of prospective license holders. The present study examined this possibility by analyzing data from Missouri’s first-ever applicant pool for medical marijuana dispensaries. During the evaluation of applications in Missouri, a state-appointed scorer used a formula to assign numerical scores based on a variety of attributes possessed by prospective businesses. The official rubric scores reported from this process provided an indirect measure of dispensary applicant quality, where higher numerical scores were interpreted as an indication of higher quality.

## Background on medical marijuana in Missouri

The legalization of medical marijuana in Missouri was approved by voters in a referendum during the November 2018 general election. The proposal, Constitutional Amendment 2, passed after receiving approval from 65.6% of voters (Missouri Secretary of State 2018b). This amendment granted Missouri residents, who suffered from a chronic illness or health problem, the right to use medical marijuana under the supervision of a state-licensed physician (Missouri Secretary of State 2018a). Additionally, the amendment granted regulatory authority to the Missouri Department of Health and Senior Services (MODHSS) for the purpose of ensuring product accessibility and safety as well as legal compliance among qualified patients, primary caregivers, physicians, and businesses. MODHSS had the responsibility of overseeing the development of a legal market, which required the creation of an application process for allocating business licenses to grow, process, or sell medical marijuana products.

Although Constitutional Amendment 2 became effective in December 2018, legal sales of medical marijuana did not occur until October 2020. MODHSS began accepting applications for business licenses in August 2019 with a final deadline on December 31, 2019. As required by state law, a numerical scoring system was developed to assess the quality of applicants. The evaluation criteria considered many attributes of the prospective businesses, which included (1) the suitability of an applicant’s resources for operating a dispensary, (2) business plan feasibility (3) on-site security, (4) potential economic impact, (5) personal character, (6) relevant experience, and (7) ability to compete in the marketplace. Rather than having state employees evaluate applications, an independent scorer was hired by MODHSS to review applications and assign numerical scores.^3^

In an initial phase of applicant evaluations, a blind assessment utilized a scoring rubric to assign a numerical score with a maximum of 1,900 possible points. After this phase, some applicants received bonus points based on the socioeconomic conditions near a proposed dispensary or receiving the highest score in their district of the Missouri State House of Representatives.^4^ State law guaranteed that each of Missouri’s eight US congressional districts would have 24 dispensary applications approved for licenses. In each US congressional district, the 24 top-ranked applicants with the highest application scores were awarded a license, conditional on meeting all other minimum qualifications for licensure. If a top-scoring applicant failed to meet any minimum requirements, such as in-state residency among a majority of owners or a criminal background check without any disqualifying felony offenses, a license would be awarded to the applicant with the next highest score outside of the top 24. All final decisions on license applications were announced by MODHSS in January 2020.

One advantage of focusing the analysis on Missouri was a provision in the state law that barred local governments from prohibiting marijuana dispensaries.^5^ Had local prohibitions been allowed in Missouri, as other state governments have authorized (Dilley et al. 2017; Wexler 2023), it could have discouraged dispensary applications in jurisdictions that were more likely to pass them. In addition, an analysis of Missouri’s initial roll-out of medical marijuana in 2019–2020 can provide insight into the retail environment for recreational marijuana, which was later legalized by the state in December 2022. When Missouri legalized recreational use, a medical dispensary was allowed to convert its medical-only license into a comprehensive license permitting the sale of both medical and recreational marijuana. Conversion requests were approved as long as the medical dispensary was in “good standing” with MODHSS, meaning its license was not suspended, revoked, or inactive (MODHSS, 2023b). Since the state continued to maintain a limit on the aggregate number of retail outlets, licensed medical dispensaries had the first opportunity to enter the legal market for recreational cannabis.

## Literature review

Geographic variation in the quality of dispensary applicants may contribute to substate patterns in legal product sales and regulatory noncompliance. While prior literature has not examined these possibilities, the relationship between local characteristics and the location sites of marijuana businesses has received considerable attention. Many analyses of this relationship have focused on the influence of socioeconomic conditions. Some studies have observed higher densities of marijuana businesses in areas with more socioeconomic deprivation (Amiri et al. 2019; Matthay et al., 2022b; Morrison et al. 2014; Shi et al. 2016; Tabb et al. 2018), yet other studies have found this connection to be nonexistent (Boggess et al. 2014; Novak et al. 2021; Thomas and Freisthler 2016). If poorer neighborhoods are more conducive for running a marijuana dispensary, whether it is due to lower operating costs or other factors, the pool of applicants seeking state licenses to locate in these neighborhoods should include a mix of both high- and low-ranked applicants as measured by their relative scores on a state’s scoring rubric.

Dispensary owners’ preferences for lower income areas could develop due to multiple factors. As discussed by Boggess et al. (2014), one hypothesis can assume that commercial marijuana activities are perceived as local unwanted land uses (LULUs), which neighborhood residents prefer to avoid. Under this assumption, marijuana businesses may disproportionately locate in poorer communities that lack the financial resources or political influence to challenge them by enacting local ordinances or zoning regulations. This explanation would align with prior analyses of local prohibitions on marijuana businesses, which found a higher likelihood of adopting a ban among communities with higher income levels and lower proportions of racial minorities (Matthay et al., 2022a; Matthay et al., 2022b; Moiseeva 2023; Yaskewich 2022; Yaskewich 2023). However, alternative hypotheses suggest that socioeconomic deprivation still may attract retail outlets even if they are not perceived as LULUs. Dispensary owners may desire location sites near communities with cheaper real estate, lower tax burdens, or higher volumes of retail activity, which may correlate with local socioeconomic conditions.

Contrary to earlier studies, Cunningham et al. (2022) observed that poorer neighborhoods had less accessibility to medical marijuana services in New York State. Their analysis included two unique features that separated it from other literature. One feature was its analysis of medical marijuana certifying providers, which certified patient eligibility to receive prescriptions. According to their results, certifying providers were more likely to exist in communities with more college graduates, fewer Black residents, and an urban classification. Communities with a dispensary also had higher densities of college graduates compared to those without a dispensary. The other unique feature of their study was the low number of dispensary licenses distributed in New York. At the time of their study, the state only had 38 licensed dispensaries, which had located in fewer than 1% of New York’s census tracts.

A plausible explanation for the mixed findings on location decisions could involve cross-state disparities in the strictness of marijuana regulations. Wang and Wilson (2022) observed substantial variation in licensing requirements across states, which can include application and license fees, minimum levels of financial capital, or in-state residency among the principal owners. Specific to medical marijuana, state regulations also have differed according to their “medicalization,” or resemblance to regulations on pharmaceutical medications (Richard et al., 2021). Since prior studies on location decisions were based on state-specific analyses of licensed dispensaries, it is possible that the estimated effects of neighborhood characteristics were confounded by the regulatory environment. In contrast, an analysis of applicant pool data would provide a more complete understanding of how the state licensing process prevents low-quality applicants from opening dispensaries in certain neighborhoods. The absence of a licensed dispensary may occur if a neighborhood only attracted applicants who received low evaluation scores. Likewise, neighborhoods with multiple dispensaries also may have been the intended location for many rejected applicants. Due to state-level variation in the strictness of licensing requirements, prior studies on licensed-dispensary locations may not reflect spatial patterns in applicant quality, especially in states that imposed low license quotas.

The ongoing presence of unlicensed sellers provides further rationale for assessing the supply-side of legal marijuana with a sample that extends beyond licensed firms. Prior studies on unlicensed outlets have suggested that they have a propensity to locate in neighborhoods with certain attributes. In their analysis of retail outlets throughout California during its first year of recreational marijuana sales, Unger et al. (2020) found that minority communities were more likely to have only unlicensed, and no licensed, retailers. A later study by Firth et al. (2022) examined the spatial distribution of marijuana outlets within Los Angeles County, California following state efforts to crackdown on unlicensed retailers. According to their results, unlicensed retail outlets were more common in communities with more Hispanic and low-income residents whereas licensed outlets were more common in areas with higher proportions of White and college-educated residents.

Observable patterns in the locations of licensed vs. unlicensed retailers may reflect local variation in the viability of state license applicants. If limitations in their financial resources or managerial expertise cause less efficient applicants to prefer certain site locations, geographic patterns may develop among the proposed sites for accepted vs. rejected applicants. Variation in numerical scores assigned to applications may be partly explained by disparities in neighborhood demographic and economic characteristics. This paper extended the literature on the economic geography of marijuana businesses by assessing whether neighborhood characteristics had an association with scores received on dispensary license applications in Missouri.

## Methods

The analysis of application scores utilized multiple data sources. A complete roster of license applicants in Missouri was obtained from the office of MODHSS (2020), which covered all applications submitted by December 31, 2019. The applicant roster included the physical addresses of proposed dispensary sites, overall evaluation scores, and separate scores from seven subsections of the application. The neighborhood of a proposed site was identified by mapping the physical address listed in the roster to its corresponding census tract. This geocoding process was conducted using Geocodio (2023), which is an online API program. The census tract was used as the unit of measurement for neighborhood-level variables given their small population sizes and land areas.

The outcomes of interest in this study were the overall and section-specific scores assigned to dispensary applications. During the initial blind review of applications, an independent third-party scorer used a rubric with a maximum score of 1,900 points. Seven categories were used to assess the suitability of an applicant’s resources for operating a dispensary (380 pts.), business plan feasibility (380 pts.), on-site security (380 pts.), potential economic impact (304 pts.), personal character (228 pts.), relevant experience (152 pts.), and ability to compete in the marketplace (76 pts).^5^ Since the maximum possible points varied across the categories, all variables for evaluation scores were reported as the percentage of points earned by the applicant. Among the 1,201 dispensary applications submitted by the initial deadline on December 31, 2019, the overall evaluation scores ranged from 15.1 to 85%. All analyses of applicant evaluation scores were based on blind scores from the initial review phase. This blind score was preferred over the final adjusted score, which included bonus points based on local socioeconomic conditions and being the highest-ranked applicant within one’s district of the Missouri State House of Representatives.

The local characteristics of census tracts were obtained from the 2016–2020 American Community Survey (ACS) 5-year estimates (US Census Bureau 2022). The ACS contained data on demographic and socioeconomic characteristics, which could affect consumer demand and the attractiveness of owning a dispensary in a neighborhood. Demographic variables included an age dependency ratio, a binary indicator for having a majority Black or Hispanic neighborhood (> 50% of residents), and a binary indicator for the absence of a racial or ethnic majority among residents. The age dependency ratio was calculated as the proportion of residents under age 18 or above age 64 divided by the proportion of residents between ages 18 and 64. The population density of census tracts, defined as the number of residents per square mile, also was included as a covariate. It was calculated using population totals from the ACS and land areas from the US Census Bureau’s (2021) TIGER/Line Shapefiles.

Socioeconomic conditions were measured using the Centers for Disease Control and Prevention’s (CDC) Social Vulnerability Index. This index contained a sub-index on socioeconomic conditions that was constructed using a collection of census-tract variables from the ACS. The sub-index provided a percentile ranking of socioeconomic vulnerability that was derived from an aggregation of five percentile rankings for the proportions of residents who (1) had incomes below 150% of the federal poverty line, (2) were unemployed, (3) had a high housing cost burden, (4) lacked a high school diploma, and (5) had no health insurance.^7^ Values of the CDC’s sub-index for socioeconomic vulnerability ranged from 0 to 100, where higher values indicated weaker socioeconomic conditions.

Other measures of neighborhood characteristics included the retail employment share and a binary indicator for the presence of a K-12 public school. Retail employment shares were measured at the census-tract level and obtained from the ACS. They were calculated as the share of employed residents who worked in retail occupations, which was used as a proxy for local retail activity. The presence of a K-12 public school was expected to discourage interest from high-quality applications since the state law in Missouri prohibited marijuana businesses from locating near them. A roster of K-12 schools and their locations was obtained from the Missouri Department of Elementary and Secondary Education (2023).

Given the percentage format of application scores, a fractional logistic regression model was used to assess whether census-tract-level indicators of applicant quality varied according to neighborhood characteristics. This empirical approach used a logistic cumulative density function to generate predicted values between 0 and 1. A key advantage of fractional regression models, as developed by Papke and Wooldridge (1996), includes their use of quasi-maximum likelihood estimation to obtain parameter estimates when the dependent variable has fractional values and it is also possible to observe values equal to the boundaries of 0 or 1. A shortcoming of using linear models in this study would be the assumption of a constant relationship between neighborhood characteristics and application scores across all values of the explanatory variables. While the log transformation of an odds ratio in a standard logistic model would account for non-linear relationships, it would not be suitable if a dispensary applicant received a score of 0% or 100% on any of the subsections of the application.

Since many census tracts contained two or more proposed dispensary sites, more than one census-tract-level measure of applicant quality was used in the analysis. Quality measures included the median, minimum, and maximum application scores among all proposed dispensaries within the census tract, which were each analyzed using fractional logistic regression. In cases where a census tract only had one applicant, each of these values would be same. Additional analyses in this study examined the propensity to attract applicants from the bottom and top ends of the statewide distribution of scores. For these analyses, binary logistic regression models were used to estimate the likelihood of attracting proposals from the bottom quartile, top quartile, and group of winning applicants who received dispensary licenses. All empirical models in this study were estimated using the statistical software program STATA 18.0 (StataCorp 2023).

## Results

**Fig. 1:**
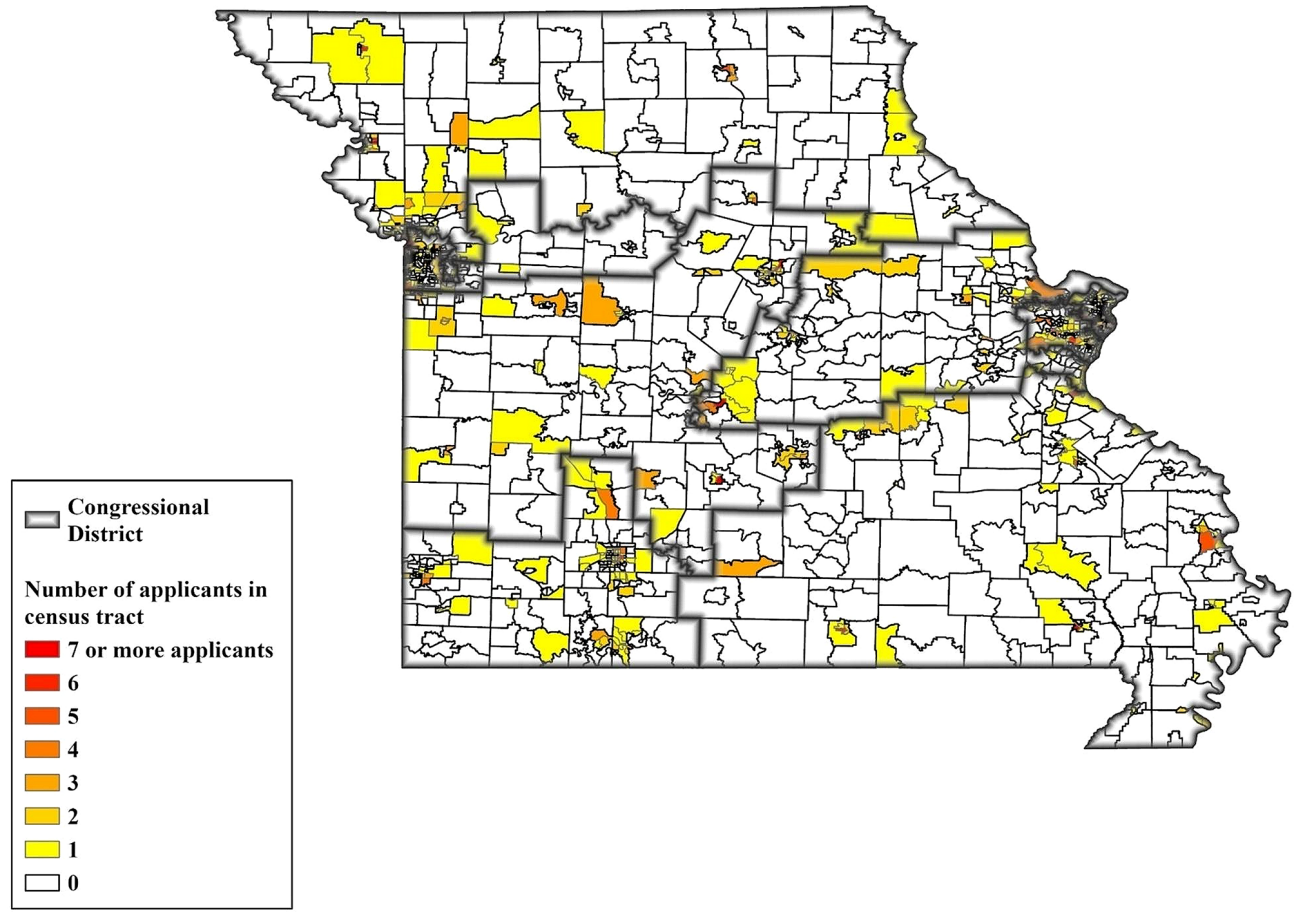
Map of Missouri census tracts where at least one dispensary site was proposed

Out of the 1,654 census tracts in Missouri, a total of 606 (or 36.6%) contained the physical location of at least one proposed site for a marijuana dispensary during the state’s review of applications. While 1,201 dispensary applications were submitted to MODHSS, only 192 licenses (or 16%) were approved. Figure 1 displays a map of census tracts in Missouri where applications for state licenses proposed the opening of marijuana dispensaries. Out of Missouri’s 1,654 census tracts, a total of 1,048 census tracts were not included in applications whereas 606 census tracts were listed as potential sites for the pool of 1,201 applicants. For census tracts included in dispensary applications, the most common numbers of dispensary site proposals were “1” (*n* = 343) and “2” (*n* = 125). There were 263 census tracts with two or more applicants. At the upper end of the distribution, a total of 16 census tracts were included in “7 or more” applications, which included one census tract with a maximum value of 17 applicants.

Definitions for each application category and descriptive statistics for the highest scores from each census tract are provided in Table 1. Sample means were stratified according to whether or not a census tract had at least one applicant approved for a license. For the whole application, the average maximum score was 79.4% in census tracts where dispensaries were approved. In other census tracts, the average maximum score was 69.5%. Two categories that tended to generate the lowest scores among top applicants were *Security* and *Economic Impact*, which had average maximum scores of 67.7% and 70.3%, respectively, where licenses were approved. For census tracts with only rejected applicants, the average maximum scores were significantly lower with values of 52.9% for *Security* and 58.2% for *Economic Impact*. The categories with the highest scores included *Character*, *Experience*, and *Competition*. For each of these categories, the average maximum score exceeded 90%.

**Table 1:** Descriptive statistics for maximum application scores in census tracts and category definitions (*n* = 606 census tracts)

	All denied	1 or more approved	
Variables	Mean (S.D)	Mean (S.D.)	Definitions and weights for application categories
Total Score (0–100%)	69.496 (10.630)	79.401** (2.152)	The overall percentage score that a dispensary applicant received on the Missouri application for a medical marijuana license. [Weight = 100%]
Dispensary Supplement (0–100%)	68.100 (12.053)	78.176** (5.53)	Questions focused on fundamental issues for operating a dispensary, such as the suitability of the site, accessibility to patients, healthcare-related experience among dispensary staff, and the availability of physicians and pharmacists for consultations. [Weight = 20%]
Business Plan (0–100%)	67.572 (10.686)	75.912** (4.109)	Questions covered employee recruitment and training, inventory management, product pricing, marketing, accounting/fiscal controls, and budget projections. Applicants were required to provide proof of adequate financial capitalization, liability insurance coverage, and the legal right to use the facility indicated on the application. [Weight = 20%]
Security (0–100%)	52.876 (13.896)	67.714** (8.076)	Applicants provided plans for preventing theft, illegal purchase, and unlawful entry. Applicants were required to provide details about employee screening and monitoring, on-site surveillance systems, and a facility’s physical infrastructure. [Weight = 20%]
Economic Impact (0–100%)	58.237 (14.472)	70.305** (9.369)	Applicants described how a dispensary would have a positive economic impact on the local area. They also provided estimates for the number of full-time equivalent jobs and the average hourly wage during the first year of operations. [Weight = 16%]
Character (0–100%)	96.365 (5.230)	98.852** (2.048)	The trustworthiness and background of all officers and managers were assessed. Applicants provided resumes, support letters, and information about prior compliance with laws related to taxes, federally funded programs, and other business practices. [Weight = 12%]
Experience (0–100%)	91.322 (24.644)	100.000** (0.000)	This section consisted of a single essay in which the applicant described the experience of each officer or manager in the legal cannabis market. [Weight = 8%]
Competition (0–100%)	91.647 (27.700)	100.000** (0.000)	This section consisted of a single essay in which the applicant described how the dispensary will be competitive in the market for medical marijuana products. [Weight = 4%]

*Note* Sample means and standard deviations are reported for the maximum application scores across census tracts. There were 175 census tracts where at least one dispensary application was approved and 431 census tracts were all applicants were denied. Statistically significant differences in sample means across categories are denoted by * and ** for significance at the 5% and 1% levels, respectively

**Fig. 2:**
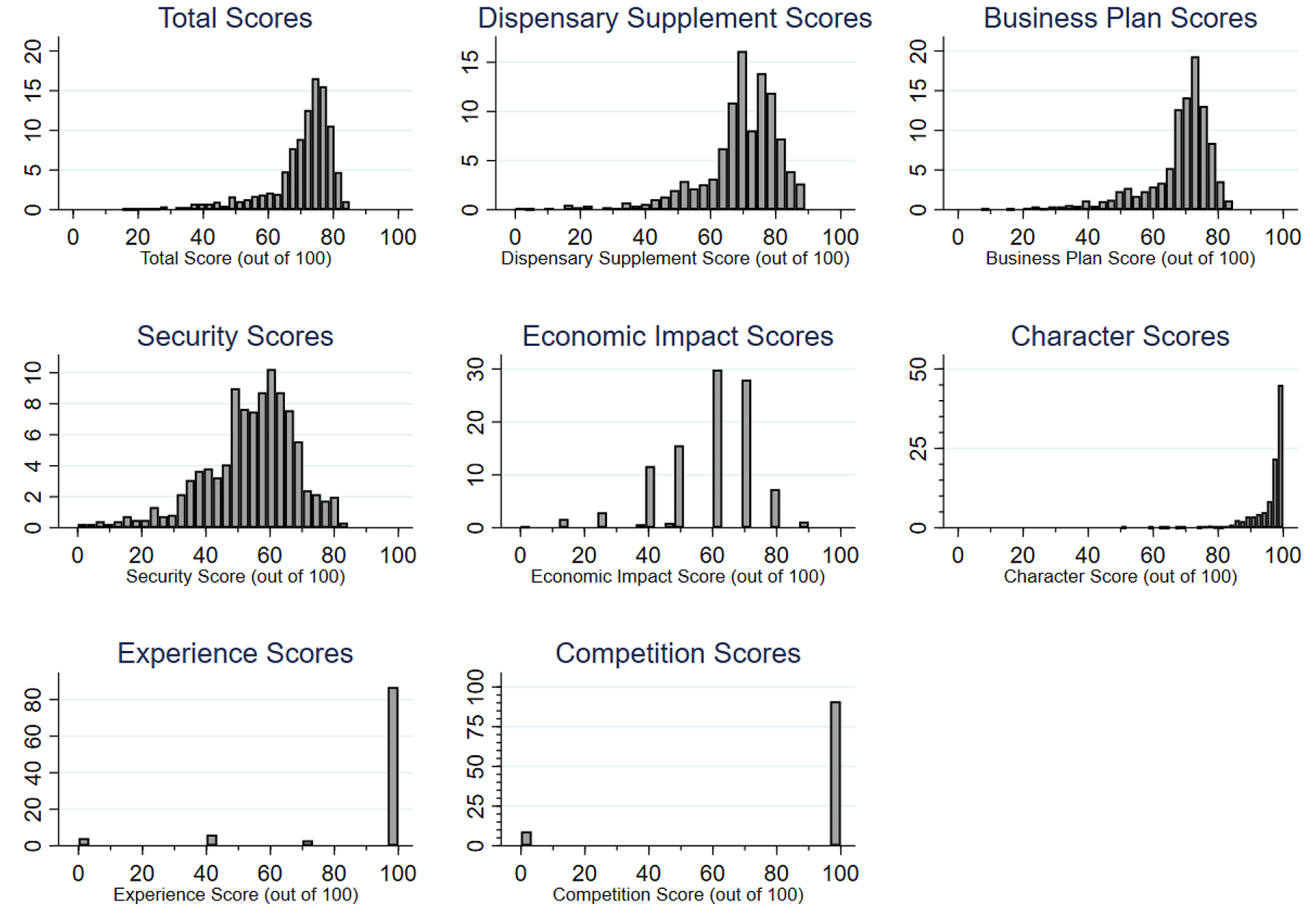
Relative frequency distributions for dispensary application scores by assessment category (*n* = 1,201 applicants).*Note* The height of each bar indicates the proportion of applicants who received the numerical evaluation score indicated on the horizontal axis

Visualizations for the entire distribution of scores among all 1,201 applicants are shown in Fig. 2. The distribution of *Total Scores*, which combined all categories, ranged from 15.1 to 85% with a median score of 73.2%. Most rubric categories resulted in a distribution of scores with considerable variation across applicants. The distribution of scores in the *Dispensary Supplement* and *Business Plan* sections each displayed a close resemblance to the distribution of *Total Scores*. However, the *Experience* and *Competition* categories generated little variation in the applicant pool as most applicants received perfect scores in both areas.

Descriptive statistics for the independent variables are shown in Table 2. The full sample consisted of the 606 census tracts that were listed as potential dispensary sites in license applications. These neighborhoods exhibited sizeable variation in population density, age dependency ratios, socioeconomic vulnerability, and retail employment shares. Approximately 85% of these census tracts had majority non-Hispanic White populations while two-thirds of the sample contained a K-12 public school within its borders. A matrix of Pearson correlation coefficients indicated that the bivariate correlations among the independent variables were unlikely to be problematic in multivariate analyses.

**Table 2:** Descriptive statistics and correlation matrix for independent variables

Variables	Mean (S.D.)	(1)	(2)	(3)	(4)	(5)	(6)	(7)
*Continuous variables*								
**(1)** Population density (in thousands)	2.444 (2.482)	1						
**(2)** Age dependency ratio x 100	63.102 (20.981)	–0.407**	1					
**(3)** Socioeconomic vulnerability index	51.353 (28.369)	0.071	–0.114**	1				
**(4)** % Retail industry employment	11.865 (5.443)	–0.181**	–0.005	0.172**	1			
*Binary variables*								
**(5)** Black or Hispanic majority (0,1)	0.101 (0.301)	0.274**	–0.015	0.403**	–0.114**	1		
**(6)** No racial or ethnic majority (0, 1)	0.048 (0.214)	0.161**	–0.111**	0.110**	–0.016	–0.072	1	
**(7)** K-12 public school (0,1)	0.670 (0.471)	–0.096*	0.015	–0.000	–0.014	–0.052	–0.049	1
Sample size	606							

*Note* The statistical significance of pairwise Pearson correlation coefficients are denoted by * and ** for significance at the 5% and 1% levels, respectively

Estimates of fractional and binary logistic regression models for *Total Scores* are shown in Table 3. For ease of interpretation, fractional model estimates of average marginal effects are reported as semi-elasticity coefficients. These coefficients are interpreted as the percentage point change in overall application scores associated with a 1% change in a continuous independent variable. Odds ratios are reported for the binary logistic models. The results indicated that neighborhood characteristics affected patterns in scores across census tracts, but they did not alter the odds of attracting a winning dispensary proposal. Neighborhoods with higher population densities tended to attract higher-scoring applicants. On average, a 1% increase in population density was associated with a 1.1% pt. increase in the median score in a census tract. The lowest and highest scores in census tracts also were positively significantly correlated with population density.

**Table 3:** Regression model estimates for dispensary application scores across census tracts

	(1) Median Total Score (0-100%)	(2) Lowest Total Score (0-100%)	(3) Highest Total Score (0-100%)	(4) Application in Bottom Quartile (0,1)	(5) Application in Top Quartile (0,1)	(6) Application Awarded License (0,1)
	Frac. Logistic	Frac. Logistic	Frac. Logistic	Logistic	Logistic	Logistic
***Independent variables***	Marginal Effect [95% C.I.]	Marginal Effect [95% C.I.]	Marginal Effect [95% C.I.]	Odds Ratio [95% C.I.]	Odd Ratio [95% C.I.]	Odds Ratio [95% C.I.]
Continuous variables	Semi-elasticities: (ΔScore)/(%ΔX)			Odds Ratios: (ΔOdds)/(ΔX)		
Population density(in thousands)	0.011** [0.003, 0.018]	0.014** [0.004, 0.024]	0.010* [0.002, 0.017]	0.939 [0.867, 1.016]	1.048 [0.967, 1.135]	0.969 [0.882, 1.065]
Age dependency ratio x 100	–0.004 [–0.028, 0.020]	0.027 [–0.006, 0.059]	–0.007 [–0.034, 0.019]	0.995 [0.986, 1.003]	1.000 [0.991, 1.009]	1.009 [1.000, 1.019]
Socioeconomic vulnerability index	–0.013 [–0.030, 0.005]	–0.027* [–0.048, − 0.005]	–0.005 [–0.023, 0.013]	1.008* [1.002, 1.015]	1.002 [0.995, 1.009]	1.002 [0.995, 1.009]
% Retail industry employment	0.002 [–0.015, 0.020]	0.002 [–0.020, 0.025]	0.003 [–0.014, 0.020]	0.992 [0.962, 1.024]	0.998 [0.966, 1.031]	0.993 [0.959, 1.029]
Binary variables	Level-level effects: (ΔScore)/(ΔX)			Odds Ratios: (ΔOdds)/(ΔX)		
Black or Hispanic majority (0,1)	0.005 [–0.021, 0.031]	0.028 [–0.005, 0.062]	–0.007 [–0.035, 0.021]	0.584 [0.319, 1.071]	0.450** [0.227, 0.891]	0.655 [0.307, 1.401]
No racial or ethnic majority (0, 1)	0.032** [0.010, 0.054]	0.046* [0.009, 0.083]	0.020 [–0.004, 0.044]	0.448* [0.189, 1.069]	0.890 [0.387, 2.045]	1.289 [0.518, 3.208]
K-12 public school (0,1)	–0.003 [–0.018, 0.013]	0.002 [–0.019, 0.022]	–0.002 [–0.018, 0.014]	0.891 [0.632, 1.256]	1.284 [0.899, 1.836]	1.039 [0.708, 1.524]
Wald χ^2^	24.77**	26.33**	14.80*	14.40*	8.25	8.74
p-value (Prob. > χ^2^)	0.001	0.000	0.039	0.045	0.311	0.272
Sample size	606	606	606	606	606	606

*Note* Average marginal effects and confidence intervals are reported for fractional logistic models. For continuous variables, semi-elasticity estimates indicate the unit change in the predicted outcome given a 1% change in X. For categorical X variables, the marginal effects are shown in level-level form indicating the unit change in the predicted outcome given a one-unit change in X. Odds ratios are reported for binary logistic models. Statistical significance is denoted by * and ** for significance at the 5% and 1% levels, respectively

Socioeconomic vulnerability was negatively associated with application scores but only in the analysis of minimum scores. The lowest scores in census tracts tended to fall by 2.7% pts. for each 1% increase in the socioeconomic vulnerability index. Additionally, binary logistic models estimated that each 1 pt. rise in the socioeconomic vulnerability index was associated with a 0.8% increase in the odds of attracting a dispensary proposal ranked in the bottom quartile of statewide scores. Racial and ethnic variables also were significantly correlated with application scores. Compared to majority non-Hispanic White neighborhoods, census tracts with Black or Hispanic majorities were less likely to attract a dispensary proposal that scored in the top 25% of the statewide pool. In contrast, scores tended to be higher in neighborhoods where the portions of White, Black, and Hispanic residents were each below a 50% majority.

When fractional logistic models were used to assess scores in each category of the dispensary application, neighborhood characteristics appeared to affect some categories more than others. Table 4 displays model estimates for the five categories that had continuous distributions of scores. Population density was positively associated with the median score within a census tract on the *Dispensary Supplement*, *Business Plan*, and *Security* categories. Median scores in census tracts with no racial or ethnic majority also were higher on the *Dispensary Supplement* and *Business Plan* sections as well as the *Economic Impact* category. No neighborhood-level variables were related to median scores for the *Character* category.

**Table 4:** Fractional logistic regression models for the median scores across by category

	(1) Dispensary Supplement (0-100%)	(2) Business Plan (0-100%)	(3) Security (0-100%)	(4) Economic Impact (0-100%)	(5) Character (0-100%)
***Independent variables***	Marginal Effect [95% C.I.]	Marginal Effect [95% C.I.]	Marginal Effect [95% C.I.]	Marginal Effect [95% C.I.]	Marginal Effect [95% C.I.]
Continuous variables	Semi-elasticity coefficients: (ΔScore)/(%ΔX)				
Population density (in thousands)	0.011* [0.003, 0.020]	0.012** [0.004, 0.019]	0.010* [0.001, 0.020]	0.009 [–0.002, 0.021]	0.002 [–0.002, 0.006]
Age dependency ratio x 100	0.005 [–0.021, 0.030]	–0.007 [–0.032, 0.017]	–0.007 [–0.037, 0.024]	0.005 [–0.028, 0.039]	0.004 [–0.009, 0.017]
Socioeconomic vulnerability index	–0.018 [–0.038, 0.002]	–0.010 [–0.027, 0.007]	–0.010 [–0.032, 0.013]	–0.015 [–0.038, 0.008]	–0.003 [–0.012, 0.006]
% Retail industry employment	0.002 [– 0.017, 0.021]	0.002 [–0.017, 0.020]	0.003 [–0.019, 0.025]	0.007 [–0.016, 0.029]	0.002 [–0.007, 0.011]
Binary variables	Level-level effects: ΔPr(Y = 1)/(ΔX)				
Black or Hispanic majority (0,1)	0.022 [–0.003, 0.048]	0.007 [–0.019, 0.032]	–0.009 [–0.041, 0.024]	0.010 [–0.029, 0.049]	–0.005 [–0.018, 0.009]
No racial or ethnic majority (0, 1)	0.026* [0.004, 0.048]	0.035** [0.012, 0.059]	0.034 [–0.006, 0.075]	0.053** [0.015, 0.090]	0.006 [–0.009, 0.020]
K-12 public school (0,1)	–0.001 [–0.018, 0.017]	–0.005 [–0.020, 0.010]	–0.004 [–0.024, 0.017]	0.000 [–0.021, 0.021]	–0.003 [–0.011, 0.005]
Wald χ^2^	18.59**	27.42**	11.39	13.56	4.38
p-value (Prob. > χ^2^)	0.010	0.000	0.122	0.060	0.735
Sample size	606	606	606	606	606

*Note* Average marginal effects and confidence intervals are reported for fractional logistic models. For continuous variables, semi-elasticity estimates indicate the unit change in the predicted outcome given a 1% change in X. For categorical X variables, the marginal effects are shown in level-level form indicating the unit change in the predicted outcome given a one-unit change in X. Statistical significance is denoted by * and ** for significance at the 5% and 1% levels, respectively

Neighborhood characteristics also appeared relevant in attracting applicants from the bottom quartile of statewide category scores. Binary logistic models for attracting at least one application from the bottom quartile of a category are provided in Table 5. On average, there was a lower likelihood of attracting low performers on the *Dispensary Supplement* among census tracts with higher population densities, larger age dependency ratios, and better socioeconomic conditions. Poorer socioeconomic conditions tended to attract low performers on the *Dispensary Supplement*, *Business Plan*, and *Security* categories, but not the *Economic Impact* or *Character* categories. The only variable with a statistically significant relationship with outcomes in the *Character* category was the age dependency ratio, which was inversely related to attracting a low-scoring applicant. A neighborhood’s racial and ethnic composition also exhibited significant effects for some categories. Black and Hispanic neighborhoods had 53.5% lower odds of attracting low performers on the *Economic Impact*. Neighborhoods with no majority group among residents had lower odds of attracting low performers on the *Business Plan* section by a magnitude of 59.1%.

**Table 5:** Logistic regression models for the attracting an applicant from the bottom quartile of scores in each category

	(1) Dispensary Supplement (0,1)	(2) Business Plan (0,1)	(3) Security (0,1)	(4) Economic Impact (0,1)	(5) Character (0,1)	(6) Experience Not Perfect (0,1)	(7) Competition Not Perfect (0,1)
***Independent variables***	Odds Ratio [95% C.I.]	Odds Ratio [95% C.I.]	Odds Ratio [95% C.I.]	Odds Ratio [95% C.I.]	Odds Ratio [95% C.I.]	Odds Ratio [95% C.I.]	Odds Ratio [95% C.I.]
Population density (in thousands)	0.870** [0.791, 0.957]	0.955 [0.882, 1.034]	0.930 [0.860, 1.007]	0.939 [0.860, 1.026]	0.947 [0.871, 1.030]	0.857** [0.773, 0.950]	0.894 [0.790, 1.011]
Age dependency ratio x 100	0.988 [0.978, 0.998]	0.994 [0.985, 1.004]	0.997 [0.988, 1.006]	0.992 [0.982, 1.003]	0.991* [0.982, 1.000]	0.993 [0.982, 1.004]	0.990 [0.978, 1.003]
Socioeconomic vulnerability index	1.008* [1.001, 1.015]	1.009* [1.002, 1.016]	1.008* [1.001, 1.015]	1.007 [0.999, 1.014]	1.004 [0.997, 1.011]	1.006 [0.998, 1.015]	1.010* [1.001, 1.020]
% Retail industry employment	0.996 [0.964, 1.029]	0.994 [0.963, 1.027]	0.998 [0.967, 1.030]	0.994 [0.960, 1.028]	1.011 [0.978, 1.044]	1.009 [0.975, 1.045]	0.997 [0.956, 1.038]
Black or Hispanic majority (0,1)	0.756 [0.403, 1.420]	0.683 [0.368, 1.268]	0.629 [0.328, 1.203]	0.465* [0.226, 0.955]	1.308 [0.702, 2.435]	0.801 [0.378, 1.701]	0.603 [0.239, 1.520]
No racial or ethnic majority (0, 1)	0.503 [0.185, 1.371]	0.409* [0.152, 1.099]	0.889 [0.366, 2.158]	0.620 [0.235, 1.637]	1.307 [0.547, 3.124]	0.678 [0.216, 2.126]	0.409 [0.087, 1.915]
K-12 public school (0,1)	1.075 [0.749, 1.542]	1.092 [0.766, 1.557]	0.879 [0.616, 1.254]	1.032 [0.706, 1.509]	1.109 [0.778, 1.582]	1.098 [0.725, 1.663]	0.889 [0.554, 1.426]
Wald χ^2^	20.83**	13.23	11.89	12.97	9.39	18.01*	15.72*
p-value (Prob. > χ^2^)	0.004	0.067	0.104	0.073	0.226	0.012	0.028
Sample size	606	606	606	606	606	606	606

*Note* Odds ratios and confidence intervals are reported for binary logistic regression models. Statistical significance is denoted by * and ** for significance at the 5% and 1% levels, respectively

Since most applicants received perfect scores of 100% on the *Experience* and *Competition* sections, columns 6 and 7 of Table 5 report estimates for logistic models that used a different indicator of a low-performing applicant. Instead of the bottom quartile, the dependent variable equaled “1” if at least one dispensary proposal in a census tract did not receive a perfect category score. According to the results, densely populated communities were less likely to attract a site proposal that lacked a perfect score on the *Experience* section. This finding suggests that medical dispensary applicants were more likely to target sites in densely populated areas when the principal owners possessed more relevant experience in the legal cannabis market. Meanwhile, socioeconomic vulnerability made census tracts more likely to attract applicants without perfect *Competition* scores. Lower scores in this category indicated that applicants exhibited a lower aptitude for being able to compete with other dispensaries. Compared to more prosperous areas, poorer census tracts tended to attract businesses with weaker signals of their ability to compete.

In Table 6, binary logistic models were estimated for attracting applicants from the top quartile of each category, which can be seen in columns 1 through 5. Compared to the findings for the bottom quartile, local characteristics were less effective in explaining location patterns among high-scoring applicants. The models were statistically insignificant for all categories except for the *Business Plan* category. In the *Business Plan* model, top-quartile applicants only appeared to prefer densely populated communities as sites for their proposed dispensaries. The other independent variables had no effect on attracting top applicants. Column 6 displays results of a binary logistic regression model indicating whether any applicant from the census tract was approved for a license. For each of the neighborhood characteristics, the estimated effects on the odds of receiving a license were statistically insignificant.

**Table 6:** Logistic regression models for attracting an applicant from the top quartile of scores in each category

	(1) Dispensary Supplement (0,1)	(2) Business Plan (0,1)	(3) Security (0,1)	(4) Economic Impact (0,1)	(5) Character (0,1)	(6) License Approved (0,1)
***Independent variables***	Odds Ratio [95% C.I.]	Odds Ratio [95% C.I.]	Odds Ratio [95% C.I.]	Odds Ratio [95% C.I.]	Odds Ratio [95% C.I.]	Odds Ratio [95% C.I.]
Population density (in thousands)	0.980 [0.905, 1.062]	1.140** [1.049, 1.238]	0.998 [0.920, 1.083]	1.027 [0.919, 1.149]	1.060 [0.980, 1.147]	0.969 [0.882, 1.064]
Age dependency ratio x 100	1.003 [0.994, 1.012]	1.000 [0.990, 1.009]	0.996 [0.987, 1.005]	1.000 [0.986, 1.014]	0.997 [0.988, 1.007]	1.009 [1.000, 1.019]
Socioeconomic vulnerability index	0.997 [0.991, 1.004]	1.001 [0.994, 1.007]	1.001 [0.995, 1.008]	1.004 [0.994, 1.013]	0.999 [0.992, 1.006]	1.002 [0.995, 1.029]
% Retail industry employment	0.992 [0.959, 1.026]	1.017 [0.985, 1.051]	1.016 [0.984, 1.049]	1.003 [0.961, 1.048]	0.999 [0.965, 1.034]	0.993 [0.959, 1.029]
Black or Hispanic majority (0,1)	1.079 [0.576, 2.020]	0.676 [0.351, 1.300]	0.585 [0.298, 1.146]	0.964 [0.392, 2.374]	0.955 [0.490, 1.862]	0.655 [0.307, 1.401]
No racial or ethnic majority (0, 1)	1.041 [0.434, 2.499]	1.374 [0.599, 3.152]	1.166 [0.514, 2.649]	0.956 [0.321, 2.846]	1.509 [0.650, 3.504]	1.289 [0.518, 3.208]
K-12 public school (0,1)	1.332 [0.222, 1.438]	1.385 [0.962, 1.992]	1.089 [0.763, 1.555]	1.036 [0.638, 1.681]	0.995 [0.689, 1.438]	1.039 [0.708, 1.524]
Wald χ^2^	5.05	17.15*	6.01	1.11	5.89	8.84
p-value (Prob. > χ^2^)	0.654	0.017	0.538	0.993	0.552	0.272
Sample size	606	606	606	606	606	606

*Note* Odds ratios and confidence intervals are reported for binary logistic regression models. Statistical significance is denoted by * and ** for significance at the 5% and 1% levels, respectively

## Discussion and conclusion

The main results of this study provide insight into how a state’s review of dispensary license applications can influence the supply-side of legal markets. Fractional logistic regression models indicated that applicant evaluation scores varied according to neighborhood characteristics. When census-tract-level measures of applicant quality were analyzed, scores tended to be better in areas with high population densities. Socioeconomic vulnerability also was correlated with application scores, but it primarily was associated with a greater likelihood of attracting site proposals from low-scoring applicants. These findings were stronger for the sections of license applications that covered the feasibility of business plans and on-site security, but less influential in assessments of personal character and economic impact.

The finding of higher application scores in densely populated areas could be driven by regional differences in the cost of operating a dispensary. Businesses in metropolitan areas usually experience higher labor costs, building and land prices, and other expenses compared to businesses in non-metropolitan areas. As large clusters of consumers and economies of scale in retail attract the most competitive and efficient sellers into urban markets, this cost differential may deter less efficient applicants who have fewer resources. Better application scores for proposed sites in densely populated areas can reflect self-selection based on costs as well as the relative proximity and availability of professional consultants in larger cities, who can assist with preparing license applications. Score patterns based on population density can help guide outreach efforts by state regulatory commissions, which can disseminate information and provide technical assistance to prospective applicants from underserved areas.

Lower application scores also were observed in communities with poorer socioeconomic conditions. In addition, majority Black and Hispanic neighborhoods were less likely to attract applicants from the top quartile of the statewide applicant pool. These results contribute to existing literature on cross-neighborhood variation in marijuana outlet availability. Some earlier studies have observed higher densities of marijuana businesses in poorer areas (Amiri et al. 2019; Matthay et al., 2022b; Morrison et al. 2014; Shi et al. 2016; Tabb et al. 2018), whereas other studies have not (Boggess et al. 2014; Novak et al. 2021; Thomas and Freisthler 2016). One implication from this collection of results is the potential role of a state’s licensing laws in reducing inequitable access to legal markets. Geographic inequities may be greater in states with looser restrictions on licenses, such as those concerning statewide license quotas, minimum levels of financial capital, or mandatory license fees.

The finding of nonrandom neighborhood patterns in application scores invites further examination into their predictive validity for future outcomes. While neighborhood characteristics were correlated with scores on the *Business Plan* and *Dispensary Supplement* sections of Missouri’s dispensary license application, it is not certain whether higher scores were associated with a licensed dispensary’s future profitability or compliance with state regulations. Past studies have found high rates of compliance with customer identification checks and the minimum customer age among licensed outlets (Buller et al. 2019; Fell et al. 2022; Lenk et al. 2021) whereas unlicensed sellers have shown higher rates of noncompliance with regulations (Nicholas et al. 2021). However, extant literature has given less attention to relatively complex activities where incidents of noncompliance are more common, such as financial reporting and seed-to-sale inventory tracking. If the evaluation rubric has predictive validity, the likelihood of committing these legal infractions should be correlated with dispensary application scores. Similarly, future work should investigate whether application scores correlate with other performance outcomes, such as customer satisfaction, revenue growth, or avoiding closure.

Despite the contributions of this paper, any conclusions made from the results must consider limitations in the research design. The analysis of applicant data was limited to a single state and only included applications for medical marijuana dispensaries. It is possible that disparities in application requirements and the maximum number of licenses in other states could lead to different outcomes. State regulations and the number of interested applicants may differ substantially when commercial licenses are distributed for recreational marijuana. The leniency of regulations also can evolve years after the initial legalization of marijuana. Analyses of longitudinal data are needed to understand if locational patterns in outcomes are similar for license applications submitted in later years.

Methods of license allocation also can vary across states with dispensary quotas. In Missouri’s 2019–2020 application process for medical marijuana, licenses were awarded to the 24 highest-scoring applicants in each congressional district. Alternatively, other states have used a random lottery to ration licenses among eligible applicants who meet a minimum score, which may alter applicant behavior and the distribution of scores. For several states, regulatory commission decisions on license applications have been followed by formal appeals and legal challenges from rejected applicants, who often make accusations of a flawed process (Associated Press 2023; Erickson 2020; Twedt 2018). After Missouri’s initial distribution of dispensary licenses, there were over 500 appeals for rejected applicants. Some applicants alleged that the process resulted in inconsistencies in scoring, a lack of transparency, and an unfair practice of awarding bonus points to specific zip codes (Hardy et al. 2020; Smith 2021). Regardless of the validity of these accusations, further analyses that utilize cross-state comparisons of application data could help policymakers identify more efficient approaches toward rubric design, applicant outreach, and decision-making processes.

### Notes

1. Goldstein and Sumner (2022) estimated that over 70% of marijuana purchased, by weight, in California was likely to occur in the illegal market. Likewise, Unger et al. (2020) identified over 600 unlicensed retail outlets in the state compared to approximately 450 licensed outlets. A later study by Firth et al. (2021) found over 100 unlicensed sellers in Los Angeles County alone.
2. Another example of cross-state disparities in licensing policies can include the outlier of Oklahoma, which has issued more dispensary licenses for medical marijuana than any other state. By May 2023, during a time when Oklahoma only legalized medical marijuana, the state had a total of 2,865 licensed dispensaries (Oklahoma Medical Marijuana Authority 2023). In comparison, the state of Missouri had 215 licensed dispensaries despite being a state that legalized both medical and adult-use marijuana (MODHSS, 2023a).
3. The vendor selected for scoring applications was Wise Health Solutions, LLC. This company is a joint venture between Oaksterdam University and Veracious Investigative & Compliance Solutions, LLC, which are both entities that specialize in the field of cannabis. According to MODHSS (2023a), all scorers had professional backgrounds that qualified them for implementing the scoring system, which included many individuals with graduate degrees at the master’s or doctoral levels.
4. When assigning bonus points to the top applicant in each State House district, MODHSS (2023a) calculated the bonus by multiplying the state average of blind scores by 5%. Bonus points based on socioeconomic conditions were calculated by multiplying the state average of blind scores on the economic development section by a zip code factor. Values for the zip code factor were either 0.0, 0.3, or 0.4 and determined based on the unemployment rate in a zip code. MODHSS used a factor of 0.4 for a zip codes categorized as having the highest unemployment rates, 0.3 for another category of zip codes with high employment rates, and 0.0 for zip codes that were not classified as having high unemployment.
5. Although Constitutional Amendment 2 prevented local governments from prohibiting marijuana businesses within their borders (Missouri Secretary of State 2018a), it did preserve some local autonomy. Local zoning ordinances were permitted to regulate the hours of operation, location site, and other business practices.
6. One of the seven evaluation categories included a supplemental category that was designed specifically for either cultivation, testing, manufacturing, or dispensary businesses. The first category listed was a supplemental category for assessing the suitability of dispensary-specific resources Questions for the other six categories did not vary according to the type of license.
7. In the CDC’s measurement of socioeconomic conditions, households that spent 30% or more of their annual income on housing were classified as having a high housing cost burden.

## Data Availability

The data that support the findings of this study are available in the Dataverse repository, 10.7910/DVN/CU9WE1.
